# A New Method for Localizing Brain Lesions

**Published:** 1897-06

**Authors:** 


### A NEW METHOD FOR LOCALIZING BRAIN LESIONS.

Mr. Robert Cox, of Shanghai, China, describes in the *British Medical Journal* for April 3, 1897, what he calls "a simple and infallible means of mapping out on the scalp the positions of any (or all) of the underlying convolutions of the cerebral cortex."

The apparatus consists of two parts:

1. A cerebro-graphometer, and

2. A diagrammatic map (on a gnomonic projection) of the external surface of the brain.

The cerebro-graphometer consists of the mechanical device known technically as "lazytongs," formed into a circle with two loops—one bearing numerals and the other letters from A to V.

"Localizing is performed as follows: Extend the instrument and apply the end of the lettered loop marked V to the occipital protuberance and the other end to the globella; then press down the loop to the scalp in the middle line and close the circle round the head so that the 10 on the numbered loop will lie on the lettered loop. Consult the chart for the bearings and place the letter 10 on the letter of longitude, when the number of latitude will rest over the part sought for. This instrument is equally applicable to all sized heads, and forms its own unit of measurement for each."

